# Stress, perceived competence and guilt as predictors of depression in parents with chronic pain

**DOI:** 10.3389/fpsyg.2024.1473955

**Published:** 2025-01-09

**Authors:** Irene J. Muñoz-Peña, José L. González-Gutiérrez, Laura Yunta-Rua, Juan C. Pacho-Hernández, Almudena López-López

**Affiliations:** Department of Psychology, Rey Juan Carlos University, Alcorcón, Spain

**Keywords:** parenting, parenthood, chronic pain, depression, stress, perceived competence, guilt

## Abstract

**Introduction:**

Suffering from chronic pain (CP) and coping with parenthood can be challenging for parental mental health. Pain can hinder the ability to deal with demands related to parenthood, which can negatively affect their psychological well-being because of unmet caregiving expectations.

**Methods:**

Considering the limited amount of research regarding the mental health of parents with CP, the study’s main aim was to test a predictive model based on previous scientific literature, using structural equation analysis, in which parental competence and parental guilt partially mediate the relationship between parental stress and depression. To examine the moderating role of CP, the model was tested on a group of parents with CP and a control group of parents without CP. The study included 380 parents from all over Spain, of which 200 formed the group with CP and 180 participants formed the control group. A cross-sectional design was used to collect data through self-report measures.

**Results:**

Higher levels of stress, guilt, and depression were observed in parents with CP. Based on the results, both groups of parents showed a good fit with the predictive model; parental stress was a good predictor of symptoms of depression both in parents with and without CP, parental competence mediated the relationship between parental stress and depression, being the relationship between competence and depression partially mediated by parental guilt.

**Discussion:**

This study is the first to quantitatively examine parental competence and guilt in parents with CP, and to analyze their role as mediators between parental stress and depression in both CP and healthy parents. The results confirm previous qualitative findings and extend them to parents with CP, showing that the tested model aligns with the main theories on stress, self-efficacy, and depression, as well as existing literature on CP. These results suggest the relevance of addressing parental stress levels for reducing and preventing depressive symptoms in parents with CP and the importance of working on guilt reduction and enhancing competence in order to improve the emotional well-being of parents. The need to take into account the mental health of parents with CP to improve their quality of life is discussed.

## 1 Introduction

Chronic pain (CP) is recognized as a public health problem that affects approximately 20% of the worldwide population and nearly 17% of the Spanish population (Dueñas et al., 2016; Yong et al., 2022). CP is associated with significant economic and social costs and reduced physical, psychological and social functioning (Mills et al., 2019; Yong et al., 2022). Research has shown that CP is commonly associated with difficulties related to daily activities, work, family functioning and social relationships (Cáceres-Matos et al., 2020; Saito et al., 2023; Wilson and Fales, 2015).

As rewarding as being a parent can be, suffering from CP and coping with parenthood can be challenging. As a result of parenthood, a new set of demands arise that did not exist before the child arrived, which are maintained and change over time. Parenting is a demanding and, sometimes, stressful experience and some studies indicate that parents with CP are at a greater risk of developing depressive symptoms (compared with parents without CP or CP patients who are not parents) (Dany et al., 2016; Fussner et al., 2018). The transition to parenthood and the coping mechanisms involved have been widely studied in the general population, but research focused on the emotional well-being of parents with CP is scarce.

Deater-Deckard (1998) have suggested that parental stress appears when there is an imbalance between the perceptions of available resources and demands, it is different from general stress as it is directly related to parenting responsibilities and experiences. Deater-Deckard (1998) developed a parental stress model that shares similarities with the general four-component stress model of Lazarus and Folkman (1984), in it, parental stress is viewed as a complex construct that involves several dimensions: Firstly, parenthood and/or the child/children are external stressors (as a result of the child’s/children’s inherent dependence on their parents); second, parenting cognitive appraisals vary across families and cultures, as a result, stressful aspects related to children and parenting also vary (Lin et al., 2023); thirdly, parental coping mechanisms play a significant role in determining parental stress levels; finally, parental reactions to stress are measured based on parental behavior and affect which, in turn, are related to parental emotional well-being (Deater-Deckard, 1998; Johnson, 2019). In addition, Albert Bandura’s Self-efficacy theory (Bandura, 1997) maintains that self-efficacy serves as a mediator between stressful experiences and their effects, such as emotional well-being. Since self-efficacy is described by Bandura as “an individual’s belief in their ability to arrange and carry out the actions needed to accomplish specific goals” (Bandura, 1977). This concept is very similar to perceived competence and therefore could be expected to function as a mediator between stress and its emotional consequences, such as depressive symptoms.

In the field of parental stress, the construct of parental competence includes both the domains of parental self-efficacy and satisfaction with their own performance as parent and is defined as a self-evaluative variable that assesses the belief in one’s ability to perform parenting effectively (Johnston and Mash, 1989; Ngai et al., 2010). In this regard, some studies have suggested that perceived competence could be a potential mediator between stressful life events and depressive symptoms (Scannell, 2021; Singh and Upadhyay, 2019). Taking into consideration both Deater-Deckard and Bandura’s models it could be hypothesized that parental competence mediates the relationship between parental stress and emotional symptoms, such as those found in depression. In this sense, Beck’s theory of depression (Beck, 1976) proposes that negative beliefs about oneself contribute to its development and maintenance, being one of the three components of the cognitive triad, along with negative views about the future and the world. More recent developments within this framework sustain that these three components could be described as specific kinds of cognitions that involve negative self-views, and include self-blame as one of these cognitive elements (Haaga et al., 1991), in addition, the role of guilty feelings in the maintenance of depression have also been identified in previous research (Luck and Luck-Sikorski, 2020). Therefore, self-blame, guilt and other negative cognitions may act as mediators between self-efficacy and depressive symptoms.

According to the Lazarus and Folkman (1984) model, both the idiosyncratic evaluations and cognitions about the stressor and the coping resources available to the person determine the stress response. In addition, personal variables and coping resources affect self-efficacy perception (Bandura, 1977; Bandura, 1997). This is why personal context, such as the existence of any circumstance that reduces coping resources or the existence of other sources of stress, is expected to influence the parental stress response and perceived competence. In the case of parents with CP, this condition involves both an added source of stress and a weakening in their coping resources, so it would be expected that parents with CP problems show higher stress levels and lower perceived competence than parents without CP. Previous literature indicates that parents with CP use ineffective parental coping mechanisms (Evans et al., 2005). Also, a study conducted by Wilson and Fales (2015) found that parents with CP may experience difficulties in raising their children in an affective and consistent manner. Considering these results, parental stress is likely to have a significant impact on parents with CP, however, the effects of parental stress on CP parents have scarcely been studied but studies of parents with other chronic diseases (e.g., multiple sclerosis, spinal cord injury, cancer etc.) (Pastor-Bédard et al., 2023; Turney and Hardie, 2018) and parents of children with diseases (e.g., cerebral palsy, HIV infection…) have found high levels of parental stress (Cousino and Hazen, 2013; Pinquart, 2018).

Previous research supports associations between the variables in the aforementioned models in parents, both in general population and in parents under additional stress conditions. Parental stress has been closely associated with parental competence and guilt, since both variables are influenced by the self-perception of the results of one’s own behavior, in this case, how parenting is performed. Regarding parental competence, several studies have found that parental stress is negatively associated with perceived parental competence in the general population (Crnic and Ross, 2017; Gordo et al., 2018; Tohme and Abi-Habib, 2022). Moreover, in parents with health conditions that involve an additional source of stress, parental competence seems to be lower than in healthy parents. Research on parental competence in parents with CP is limited, but Evans et al. (2005) found reduced levels of parental competence in a sample of women with varying forms of CP and a study by Wilson and Fales (2015) suggested that the ability to rear children in an affective and constant way can be influenced by the presence of CP; furthermore, numerous studies have shown that parents with CP have some difficulties in performing physical tasks related to parenting (e.g., going shopping, taking children to school, holding a baby in their arms, playing with them…) (Evans et al., 2005; Hadi et al., 2019; Hawkey et al., 2022; Smeele et al., 2020). Additionally, in several qualitative studies, despite not explicitly discussing the concept of parental competence, parents with CP expressed that difficulties associated with pain interfere with how they wished to parent (Barlow et al., 1999; Hawkey et al., 2022; Grant et al., 2004; Mitton et al., 2007; Parton et al., 2022), and said that they have difficulties expressing warmth and affection at times when they are experiencing pain (Evans and de Souza, 2008; West et al., 2012). In addition, it has been found that parents under other prolonged stress conditions, such as other health problems, chronic illnesses (such as cancer, narcolepsy, HIV…) (Cessna et al., 2016; Nehring and Cohen, 1995) and/or having children with health issues (e.g., diabetes, autism…) (Bassi et al., 2020; Mohammadi et al., 2019) frequently experience reduced parental competence in comparison to other parents.

Parental guilt is a cognitive-emotional response that arises when parents perceive that their thoughts, feelings or behaviors as parents have caused harm to their children, leading to negative self-evaluations (Haslam et al., 2020; Sutherland, 2010). In the context of caregiving, guilt is proposed as an emotion resulting from a negative self-evaluation of one’s behavior compared to one’s ideal performance as a caregiver (Prunty and Foli, 2019). This reaction may appear when parents perceive that their upbringing does not meet their own standards or cultural expectations (Tilghman-Osborne et al., 2010). Several quantitative studies in general population show that parental guilt is positively related to parental stress (Henderson et al., 2016; McGregor, 2022; Tohme and Abi-Habib, 2022). Moreover, parental self-efficacy seems to be a factor which contributes to the development of guilt in mothers (Maclean et al., 2021) and other studies have found that lower levels of perceived competence are related to higher guilt levels in parents (Henderson et al., 2016; Tohme and Abi-Habib, 2022). Among parents in high-demand care conditions, Kuhn and Carter (2006) found a significant negative association between parenting self-efficacy and parenting-related guilt in mothers of children with Autism Spectrum Disorder. Regarding parents with CP, some qualitative studies have shown that mothers experiencing CP often feel guilty about the impact their disease may have on their children’s upbringing (Kristiansen et al., 2012; Meade et al., 2012). Despite the relevance of these variables, to the best of our knowledge, no previous quantitative studies have examined parental competence or parental guilt in a sample of parents with CP. Furthermore, the present study represents a unique contribution to the field, as it is the first study analyzing the role of parental competence and guilt as mediating factors between parental stress and depression both in CP and healthy parents.

Regarding emotional consequences of parental stress, both in general population and in parents of children with chronic health problems, parental stress has been found to be closely associated with depressive symptoms (Bassi et al., 2020; Fang et al., 2021; Mohammadi et al., 2019). As previously mentioned, self-efficacy and associated beliefs, such as competence, have been proposed as mediators between parental stress and depressive symptoms. In this sense, numerous cross-sectional and longitudinal studies have confirmed the long-theorized association between mothers’ depression and lower parenting self-efficacy beliefs (Curci et al., 2021; Fang et al., 2021; Gordo et al., 2018; Oltra-Benavent et al., 2019). Moreover, according to results from a meta-analytic review of 35 longitudinal studies (Goodman et al., 2022) lower self-efficacy may contribute to worsening depression over time in parents due to, among other causes, the impact of negative self-perceptions on the development of depression. Furthermore, several quantitative studies have shown that parental guilt is positively associated with depression (Borelli et al., 2017; Liss et al., 2013; McGregor, 2022). Due to the association between guilt, parental stress and competence found in previous literature and its aforementioned role as a mediator between self-efficacy and depressive symptoms (Haaga et al., 1991), a mediational role could be also expected in the case of parental stress.

The theoretical frameworks exposed and the data from previous studies lead us to suggest a single model which unifies the relationship between parental stress, perceived competence, guilt and depression, however, no model has been tested until now either in general population or in parents under chronic stress or health conditions. Considering the limited amount of research conducted on the mental health of parents with CP, this study has two objectives: Firstly, to test a model in which parental competence and parental guilt mediate the relationship between parental stress and depression both in parents with and without CP (moderating effect). Based on previous literature, we expect that guilt will partially mediate the association between competence and depressive symptoms. Secondly, we sought to determine whether there was a difference in parental stress, competence, guilt, and depression between the two samples; we hypothesized higher stress, guilt and depressive symptoms in parents with CP, as well as lower perceived parental competence.

## 2 Materials and methods

### 2.1 Participants

The study sample was composed of 380 mothers and fathers from all over Spain, 200 participants formed the group with CP and 180 participants formed the control group (without CP). All participants were required to have at least one child under 18 years. The parents who conformed the CP group had to have a CP diagnosis from a medical professional and their pain had to have been present for at least 3 months, according to the latest classification developed by the International Association for the study of Pain (IASP) (Treede et al., 2019). A variety of CP conditions were represented in the CP group: 106 participants with fibromyalgia (53%), 43 participants with headaches (21.5%), 27 participants with inflammatory rheumatoid pain (13.5%), 17 participants with back pain (8.5%), 3 participants with osteoarthritis (1.5%), 2 participants with other musculoskeletal pain (1%) and 2 participants with endometriosis (1%). Some participants had more than one painful condition.

The sociodemographic characteristics of both samples are shown in Table 1. The group of parents with CP included 150 mothers and 50 fathers. The control group of parents without pain was made up of 147 mothers and 33 fathers. The mean age of the CP sample and the control group were 43.01 (SD = 6.38) and 38.71 (SD = 6.25), respectively.

**Table 1:** Sociodemographic characteristics of both samples.

		Chronic pain group (*n* = 200)	Group without chronic pain (*n* = 180)
Age (mean, SD)		43.01 (SD = 6.38)	38.71 (SD = 6.25)
**Gender**			
	Male	150 (75%)	147 (81.7%)
	Female	50 (25%)	33 (18.3%)
**Educational level**			
	Primary studies	15 (7.5%)	5 (2.8%)
	Secondary studies	28 (14%)	17 (9.4%)
	Intermediate education	71 (35.5%)	34 (18.9%)
	Higher education	71 (35.5%)	83 (46.1%)
	Postgraduate studies	15 (7.5%)	41 (22.8%)
**Laboral status**			
	Employed	135 (67.5%)	147 (81.7%)
	Unemployed	65 (32.5%)	33 (18.3%)
**Marital status**			
	Married	156 (78%)	106 (58.9%)
	In relationship	16 (8%)	35 (19.5%)
	Single	6 (3%)	27 (15%)
	Separated/divorced	22 (11%)	11 (6.1%)
	Widowed	0	1 (0.5%)
**Number of children**			
	One	74 (37%)	73 (40.6%)
	Two	104 (52%)	72 (40%)
	Three	19 (9.5%)	29 (16.1%)
	Four	3 (1.5%)	3 (1.7%)
	Five	0	3 (1.7%)

SD, standard deviation; n, number of subjects.

### 2.2 Measures and instruments

#### 2.2.1 Sociodemographic variables

Participants responded to questions related to the following sociodemographic background: educational level, employment status, marital status, number of children and children’s age. Both samples were compared based on these variables. The sociodemographic characteristics of both samples are presented in Table 1.

#### 2.2.2 Parental stress

The Parental Stress Scale (PSS) (Berry and Jones, 1995) has 18 items that provide a description of the parent–child relationship and how parents feel regarding their parental role. It provides a parenting stress score that considers the positive aspects of parenting (i.e., “I feel happy in my role as a parent”) and those considered negative and stressful (i.e., I feel overwhelmed by the responsibility of being a parent”). In terms of internal consistency, the original scale is adequate (α = 0.84). This study used the Spanish adaptation, which is a 12-item version composed of Likert-type items with 5 response options (Oronoz et al., 2007).

#### 2.2.3 Parental competence

The Parenting Sense of Competence (PSOC) (Johnston and Mash, 1989) evaluates the self-perception of parental competence: how parents perceive their roles. The PSOC is one of the most commonly used and psychometrically accepted measures of perceived parenting competence (Hurley et al., 2014). The measure of parental competence can be used in its entirety or just one of its subconstructs: parental efficacy and satisfaction. The original instrument has an internal consistency of 0.79. The Spanish version adapted by Oltra-Benavent et al. (2019) was used for this investigation, which is a 17-item version with 6 response options and has good internal consistency for the scale (α = 0.85) (i.e., “I know what to do now to solve the problems of caring for my child” or “There are other things I am better at than being a mother”).

#### 2.2.4 Parental guilt

The Guilt About Parenting Scale (GAPS) (Haslam et al., 2020) is a unidimensional structured scale. In this scale, parental guilt is measured from one (very strongly disagree) to seven (very strongly agree) on a Likert scale, with higher scores indicating a greater level of guilt (i.e., “I often worry I am not as good a parent as I should be” or “I often feel it is my fault if my child gets upset”). The internal consistency for the original scale is strong (α = 0.89). This study used a Spanish version developed by a translation (English-Spanish) – back translation (Spanish-English) process of the items following the indications by Muñiz et al. (2013). This translation obtained good internal consistency indices in the study sample (α = 0.904).

#### 2.2.5 Depression

The Hospital Anxiety and Depression Scale (HADS) (Zigmond and Snaith, 1983) is a widely used self-reported screening instrument, which allows the detection of the presence of anxious and depressive disorders in non-psychiatric outpatient clinical settings. Our study used the depression scale (HADD) which has 7 Likert-type items and measures depressive symptoms during the previous week in its Spanish version by Tejero et al. (1986), which has excellent internal consistency for the HADD subscale (α = 0.84).

### 2.3 Procedure

Before starting the study, the Research Ethics Committee of the University approved its development. Online data collection software was used to collect data in a cross-sectional design. Participants were informed of the study’s purpose, the processing of the personal data, and their data protection rights before giving their informed consent. Different methods were used to recruit participants: posts on social media groups and blogs targeting parents and CP patients, university bulletin boards and national CP patient associations. A request for collaboration was made via telephone or e-mails to patient associations. To participate in the study, associations were required to sign a letter of acceptance, and then they publicized the survey through any means they considered appropriate (social networks, websites or e-mail). The study was conducted in collaboration with 36 patient associations throughout the national territory of Spain. The study used a snowball sample, which allowed participants to invite other parents who might be interested in participating.

The inclusion criteria for both groups were: (1) to be over the legal age, (2) to have at least one child under 18 years of age and (3) the CP group were required to have a diagnosis from a medical professional. The exclusion criteria for both groups were: (1) psychotic symptoms or another major psychiatric disorders, (2) severe chronic infectious, metabolic, renal, endocrine, oncological or neuromuscular diseases, (3) participation in another study that may interfere or influence the measurements and results of this study and (4) incapacitating physical or mental conditions for the understanding of the applied instruments, as well as to offer their informed consent consciously and voluntarily.

### 2.4 Statistical analysis

To examine the possible differences between the CP group and the control group in the sociodemographic variables of the parents, descriptive analyses were performed and χ^2^ and t-tests were used. An analysis of covariance (ANCOVA) was performed to compare the means of the parental variables examined (parental stress, parental competence, parental guilt and depression) and to determine whether there were any differences between the two groups. Furthermore, ANCOVA allowed for the determination that those sociodemographic factors that showed significant differences between groups, did not influence the difference in means. Additionally, the participant’s marital status was divided into two groups (with or without a partner) so that a *t*-test could be conducted to determine whether there were differences between the means of parental stress depending on whether the participants had a partner. These analyses were performed using the statistical software SPSS 25.0 (IBM Corp., Armonk, NY, United States).

**Figure 1:**
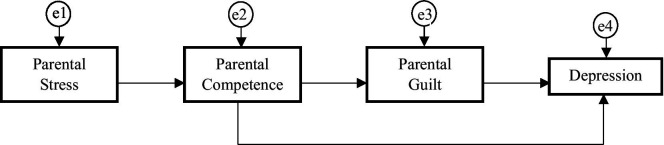
Research model.

To test the proposed mediation model (Figure 1) for both groups (CP and control), structural equation modeling (SEM) analysis was carried out using AMOS 24.0 software (IBM Corp., Armonk, NY, United States). A general model was constructed to examine if the model fit both groups. The SEM was based on four observable variables that explained the relationships. Maximum-likelihood (ML) estimation procedures were used. To test the compatibility between the developed model and the empirical data obtained, model fitness was assessed using the comparative fit index (CFI), the goodness of fit index (GFI), the Tucker-Lewis index (TLI) and the root-mean-square error of approximation (RMSEA) (Jackson et al., 2009). CFI, GFI and TLI values above 0.90, suggest the acceptability of the model and the RMSEA index of 0.06 or under suggests a close fit (Kline, 2015). The chi-square goodness-of-fit index was considered as a complementary measure, assuming a non-significant *p*-value to be a good model fit. Furthermore, to determine whether the proposed model was equivalent (i.e., invariance) between the group with CP and the group without CP, a multi-group analysis was conducted using AMOS 24.0 software (IBM Corp., Armonk, NY, United States) to detect if significant differences were present for each specific path of the model proposed (the same for both groups) (Hair et al., 2017).

## 3 Results

### 3.1 Preliminary analysis

The assumptions of the regression analyses were tested previously. First, an exploration of the data checking for the presence of univariate and multivariate outliers was performed, not based on the estimated model. Data were assessed for univariate outliers using boxplots and for multivariate outliers using a Mahalanobis Distance Test (critical value χ^2^_2_ = 13.81, *p* < 0.001), respectively (Tabachnick and Fidell, 2019). Three outliers were identified and removed. The Mardia (1970) test for multivariate kurtosis analyzed the multivariate normality of the sample which yielded statistically non-significant results (critical ratio = −1.65, *p* > 0.05), following the multivariate normality criterion.

The results of the chi-square and t-test, focused on sociodemographic variables, revealed that both groups differed significantly in the age of the parents (*t* = 6.61, *p* = 0.00), as the parents with CP showed a mean age of 43.01 (SD = 6.38) and the control parents a mean of 38.71 (SD = 6.23). Both groups also showed differences in educational level (χ^2^ = 32.77, *p* = 0.00), employment (χ^2^ = 9.94, *p* = 0.002) and marital status (χ^2^ = 33.69, *p* = 0.00), with the CP group having a lower educational level and a higher percentage of unemployed people than the control group. No statistically significant differences were found for the gender of the parents (χ^2^ = 2.47, *p* = 0.116) or the number of children (*t* = −1.035, *p* = 0.301), showing that the parents with CP had an average of 1.76 children (SD = 0.684) and the control parents an average of 1.84 (SD = 0.873). According to an additional analysis of the *t*-test conducted to determine if parental stress levels differed depending on whether parents had partners (relationship status: married or in a relationship) or not (relationship status: single, separated/divorced and widowed), no differences in stress levels were observed between participants in either the pain group (*t* = −1, *p* = 0.157) or the control group (*t* = −1.05, *p* = 0.147). In subsequent analyses focused on examining differences between groups, parents’ age, educational level, employment status and marital status were selected as covariates and controlled for.

Table 2 shows the descriptive statistics for the parents with CP (*n* = 200) and the control group (*n* = 180). The results of the univariate analysis of covariance (ANCOVA) revealed that after controlling for parents’ age, educational level, employment and marital status as covariates, parents with CP showed significantly higher levels in the variables parental stress [*F*(1, 374) = 4.11, *p* < 0.05], parental guilt [*F*(1, 374) = 16.22, *p* < 0.001] and depression [*F*(1, 374) = 25.38, *p* < 0.001], compared to the control group. Also, the CP group showed significantly lower levels of parental competence [*F*(1, 374) = 9.82, *p* < 0.001] compared to the control group. Considering the small-medium effect sizes (observed in Cohen’s d and partial eta squared), these results should be interpreted with caution (Fey et al., 2023).

**Table 2:** Descriptive statistics and ANCOVA results comparing the chronic pain group and control group in the study variables.

Measure	Chronic pain group		Control group		*F*	η_p_^2^	*d*
	*M*	SD	*M*	SD			
Parental stress	29.58	7.40	27.99	7.54	4.11*	0.01	0.15
Parental competence	66.1	10.97	70.41	11.60	9.82**	0.03	0.24
Parental guilt	48.76	13.65	43.04	13.05	16.22**	0.04	0.31
Depression	7.75	4.17	5.09	3.90	25.38**	0.06	0.38

*p < 0.05; **p < 0.001.

The clinical implications of the scores of parental stress, parental guilt and parental competence cannot be assessed since there are no cut-off points for the scales used to measure these variables. As indicated by Terol-Cantero et al. (2015), on the HADD scale (used to assess depression in this study), a score greater than 8 indicates possible cases of depression, while a score greater than 10 indicates probable cases of depression. Table 2 shows that the mean of depression for the group of parents without CP is 5.09 (SD = 3.90), whereas it is 7.75 (SD = 4.17) for the group of parents with CP. Therefore, the group of parents with CP obtained a mean that was extremely close to the high probability of depression, and considering that the standard deviation was 4.17, these results would imply that the majority of participants would exceed this threshold.

The assumption of multicollinearity was tested by examining the bivariate correlations between study variables (Table 3), having not found values greater than 0.85 and therefore no multicollinearity issues were detected.

**Table 3:** Bivariate correlations between variables.

	1	2	3	4
Parental stress (1)		−0.67**	0.45**	0.48**
Parental competence (2)	−0.66**		−0.55**	−0.59**
Parental guilt (3)	0.46**	−0.61**		0.55**
Depression (4)	0.40**	−0.54**	0.59**	

The correlations below the diagonal are from the chronic pain group whereas correlations above the diagonal are from the control group; **p < 0.01.

### 3.2 Testing the proposed model in both groups (SEM)

The path model showed good fit indices in the observed parameters. The chi-square analysis revealed a statistically non-significant *p*-value (χ^2^ = 5.30; df = 2; *p* = 0.71). As a result of its sensitivity to the sample size, this indicator should not be interpreted independently (Medrano and Muñoz-Navarro, 2017). Therefore, other standardized indices were included. The comparative fit index (CFI) showed a value of 0.995, the normalized fit index (NFI) obtained a value of 0.991 and the Tucker-Lewis index (TLI) had a value of 0.984, and all of them were excellent. In addition, an adequate value of 0.06 was obtained in the root-mean square error of approximation (RMSEA).

The model showed an excellent fit for the CP group (χ^2^ = 1.9, df = 2, *p* = 0.39, CFI = 1, NFI = 0.994, TLI = 1, RMSEA = 0.001). The final model accounted for 44% of the variance in parental competence (*R*^2^ = 0.44, *p* < 0.01), 37% of the variance in parental guilt (*R*^2^ = 0.37, *p* < 0.01) and 40% of the variance in depression (*R*^2^ = 0.40, *p* < 0.01).

**Figure 2:**
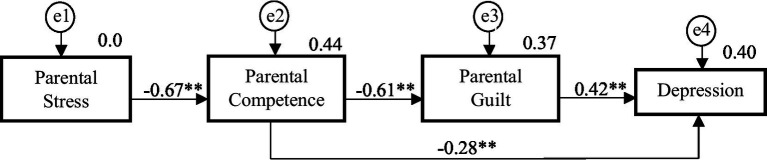
The structural model for parents with chronic pain. **p < 0.001.

Figure 2 and Table 4 show the regression weights of the CP model, showing statistically significant values for all the paths analyzed. Parental stress was negatively associated with parental competence (*p* < 0.001; *r* = −0.664). Parental competence was shown to be negatively related to both parental guilt (*p* < 0.001; *r* = −0.609) and depression (*p* < 0.001 *r* = −0.283). Parental guilt was positively associated to depression (*p* < 0.001; *r* = 0.417).

**Table 4:** The structural model for the CP group and control group.

Associations	Parents with chronic pain					Control parents				
	Regression weights				S.R.W.	Regression weights				S.R.W.
	Estimate	S.E.	C.R.	*p*	Estimate	Estimate	S.E.	C.R.	*p*	Estimate
PC ← PS	−0.99	0.08	−12.52	**	−0.67	−1.03	0.85	−12.13	**	−0.67
PG ← PC	−0.76	0.07	−10.83	**	−0.61	−0.61	0.07	−8.70	**	−0.55
D ← PG	0.13	0.02	6.02	**	0.42	0.10	0.02	4.84	**	0.33
D ← PC	−0.11	0.03	−4.08	**	−0.28	−0.14	0.02	−5.95	**	−0.41

Standardized regression weights (S.R.W.); estimation error (S.E.); critical ratio (C.R.); parental competence (PC); parental guilt (PG); parental stress (PS); depression (D). **p < 0.001.

The fit of the created model was adequate for the control group (χ^2^ = 4.95, df = 2, *p* = 0.084, CFI = 0.989, NFI = 0.982, TLI = 0.967, RMSEA = 0.091). Despite excellent values for all indices, the RMSEA value indicated that the model fit was not as good as for CP patients. According to some authors, it is possible to observe artificially high RMSEA values when the degrees of freedom are low and the sample size is small (Kenny et al., 2015). The final model accounted for 45% of the variance in parental competence (*R*^2^ = 0.45, *p* < 0.01), 30% of the variance in parental guilt (*R*^2^ = 0.30, *p* < 0.01) and 42% of the variance in depression (*R*^2^ = 0.42, *p* < 0.01).

**Figure 3:**
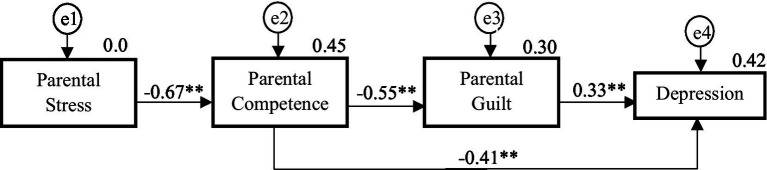
The structural model for control parents. **p < 0.001.

Figure 3 and Table 4 show the regression weights of the control group model, which, as in the case of the group of parents with CP, showed statistically significant values for all the paths analyzed. Parental stress was shown to be negatively associated with parental competence (*p* < 0.001; *r* = −0.672). Parental competence was negatively associated with both parental guilt (*p* < 0.01; *r* = −0.545) and depression (*p* < 0.001; *r* = −0.405). Parental guilt was positively related to depression (*p* < 0.001; *r* = 0.329).

### 3.3 Path analysis (multigroup moderation)—moderating effect of CP

The differences between the group of parents with CP and the group of parents without CP were tested by comparing the unconstrained and constrained models in AMOS. As the results indicated, when all structural weights were constrained, the chi-square was 10.184 and the *p*-value 0.501, indicating that the invariance of the structural weights model between the two groups was confirmed, meaning that the differences in the path coefficients between latent variables of the structural model were not significant. These results indicate that the effect of CP does not have a significant moderating effect in the structure model. To verify that CP did not influence any of the paths, two models were freely estimated except that one of the paths was constrained to be the same across the two groups (Table 5). In any of the paths examined, there were no significant differences between the CP parents’ group and those without CP, suggesting that the proposed model would be equally applicable to both groups.

**Table 5:** Results of the path comparison between both groups (CP pain and Control group).

Model comparison of the structural weights of the paths	χ	df	*p* value
Parental competence ← parental stress	0.171	1	0.679
Parental guilt ← parental competence	2.099	1	0.147
Depression ← parental guilt	0.996	1	0.318
Depression ← parental competence	0.669	1	0.413

Based on the fit for both groups examined, the proposed model was adapted satisfactorily. Nonetheless, the percentage of explained variance for the depression variable was very slightly higher in the control group (*R*^2^ = 0.42, *p* < 0.01) than in the CP group (*R*^2^ = 0.40, *p* < 0.01).

## 4 Discussion

The main aim of this study has been to test a model in which parental competence and parental guilt mediate the relationship between parental stress and depression, finding that parental guilt partially mediates the relationship between competence and depression. The model was tested in two groups: a group of CP parents and a group of control parents (i.e., controlling for CP as a moderating factor).

The SEM analysis showed a good fit of the model proposed. Moreover, the model fits similarly in both groups of parents (parents with CP and parents without CP), except regarding RMSEA. As several authors recommend not computing the RMSEA for small df models (Kenny et al., 2015), a similar fit for both groups can be considered. Furthermore, when each of the model’s paths was compared individually, no significant differences were observed between the two groups. As a result, no moderating effect of CP was observed.

Analyzed as a whole, the model tested is consistent with what would be expected based on the framework of the Lazarus and Folkman (1984) stress model, Bandura’s (1977) self-efficacy theory or Beck’s depression theory (Beck, 1976), as well as Deater-Deckard’s (1998) parental stress model. Given that personal and context-related variables do not determine, according to these models, changes in the relationships between the elements of the process, but rather in their intensity (e.g., the level of stress), it does not seem strange that the model adjusts equally in both samples and there are no changes in the weights of the trajectories, although there are differences (as will be discussed later) in the levels of the variables analyzed.

Because of cognitive-behavioral process parents experience different stress levels. According to both the Lazarus and Folkman (1984) and Deater-Deckard (1998) models, one of the main consequences of stress is the emergence of emotional alterations, such as depressive symptoms. The predictive role of parental stress over depressive symptoms is reflected in the model and supported by our data. Moreover, based on Albert Bandura’s Self-efficacy theory (Bandura, 1997) and on previous data from several studies (Scannell, 2021; Singh and Upadhyay, 2019), the tested model included perceived parenting competence as a mediator variable between parental stress and depression. Once again, our data support this role and, in addition, shows that the relationship between parental competence and depression is both direct and indirect, mediated by parental guilt. The role of guilt in this relationship is not surprising, since the lasts developments of cognitive theories of depression sustain that cognitions about the self that involve negative self-views, value judgments and self-blame are strongly linked to the development and maintenance of depression (Beck, 1976; Beck, 2008; Haaga et al., 1991; Luck and Luck-Sikorski, 2020) and those cognitive elements could predicted, in part, by self-efficacy (Passanisi et al., 2015). In this vein, the construct of parental competence is more complex than self-efficacy alone, it includes both self-efficacy and satisfaction with performance and results, having found that its self-evaluative and self-judgment components are stronger than the construct of self-efficacy (Johnston and Mash, 1989; Ngai et al., 2010). This could involve a greater predictive value of competence over guilt, and previous data supports this role both in parents in the general population (Henderson et al., 2016; Tohme and Abi-Habib, 2022) as well as in parents in highly stressful contexts (Kuhn and Carter, 2006).

Although no studies have previously tested a model that encompasses all the variables which the tested model includes, other works have analyzed the relationship between the different variables in the model separately. In this regard, our data are in coherence with previous research. In both samples analyzed, parents with a greater imbalance between their parenting skills and childcare demands (higher parental stress levels) were more likely to self-perceive a lower sense of parental competence, possibly because of feeling that their standards of childcare were not being met. This is consistent to previous studies conducted in samples of healthy parents (Oltra-Benavent et al., 2019; Tohme and Abi-Habib, 2022), and parents under stressful situations (children with disabilities, postpartum, parental mental illness, working mothers…) (Gordo et al., 2018; Kristensen et al., 2024; Kuhn and Carter, 2006; Maclean et al., 2021) in which self-rated parental stress is a strong predictor of parental competence.

A negative relationship was found between parental competence and levels of depressive symptoms in both samples (CP and control), these findings are consistent with previous research both in samples under high parental stress (Jones and Prinz, 2005; Kristensen et al., 2024; Gordo et al., 2018), as well as in samples of general parents (Curci et al., 2021; Fang et al., 2021; Oltra-Benavent et al., 2019). Individuals with lower self-efficacy may exacerbate depression by avoiding activities that could boost their sense of achievement and offer positive reinforcement (Bandura, 1977; Jacobson et al., 2001). Avoiding such behaviors reduces chances for rewarding experiences and reinforces negative beliefs about their ability to achieve important goals (e.g., being an effective parent), all of which play a role in the development and persistence of depression (Beck and Alford, 2009). Moreover, the mediating effect of parental competence observed in present data is consistent with previous studies indicating that competence, at a general and parental level, is a powerful mediator between stressful situations and depressive symptoms, suggesting that it may even serve as a protective factor against depressive symptoms (Gilmore and Cuskelly, 2008; Maciejewski et al., 2000; Philip et al., 2013; Scannell, 2021; Singh and Upadhyay, 2019).

One of the main contributions of the present study is the analysis of guilt as a mediator between parental competence and depression. Feelings of guilt are identified as a relevant predictor of depression in adults (Luck and Luck-Sikorski, 2020). In parents, McGregor’s (2022) research suggests that parental stress is closely related to parental guilt and depression and other studies have demonstrated an inverse relationship between parental competence and parental guilt in parents of general samples (Henderson et al., 2016; Tohme and Abi-Habib, 2022) and in parents in high chronically stressful contexts (Kuhn and Carter, 2006). Furthermore, parental guilt is positively associated with depressive symptoms in mothers and other parent samples (Borelli et al., 2017; Haslam et al., 2020; Liss et al., 2013) as well as in other populations of caregivers under high stress situations (caregivers, parents of children with burns and intellectual disabilities…) (Gallagher et al., 2008; Losada et al., 2018; Sveen and Willebrand, 2018). Despite of the importance that those findings seem to suggest regarding the role of guilt in the relationship between parental competence and depressive symptoms, no previous studies have analyzed its role as a mediator.

The second purpose of this study was to examine the differences between pain-affected and pain-free parents regarding all the variables included in the model: parental stress, parental competence, parental guilt and depression. ANCOVA analyses revealed that parents with CP showed significantly higher levels of parental stress and lower levels of parental competence than the control group, and higher levels of guilt and depressive symptoms. However, the effect sizes are small, and these differences should be interpreted with caution.

The higher parental stress found in parents with CP can be explained within the framework of the Lazarus and Folkman (1984) theory, since CP implies an added source of stress and, in addition, it diminishes the resources to cope with parenting demands, facilitating the increase of stress responses. Studies of parents with other chronic diseases (e.g., multiple sclerosis, spinal cord injury, cancer etc.) (Pastor-Bédard et al., 2023; Turney and Hardie, 2018) and parents of children with diseases (e.g., cerebral palsy, HIV infection…) have found high levels of parental stress (Cousino and Hazen, 2013; Pinquart, 2018), nevertheless, parental stress in CP parents has been scarcely studied and our results contrast with those found in the only other study that has analyzed stress levels among parents with CP, carried out by Johnson (2019), who found no differences between parental stress levels of parents with headache disorders and control parents. Nevertheless, the stress scores found by Johnson (2019) were abnormally low compared to the results shown in a review of 25 studies that utilized the PSS in parents (Louie et al., 2017) and this fact may explain the lack of significant differences between the groups.

Regarding differences in parental competence between parents with and without CP, our data extends previous knowledge, based on qualitative studies, to quantitative findings. The significantly lower levels of parental competence found in parents with CP (compared to the control group) are consistent with previous findings from qualitative studies that indicate many mothers who experience CP frequently experience feelings of inadequacy when performing parenting tasks, which is related to their perceived self-efficacy (Mitton et al., 2007; Wilson and Fales, 2015). Likewise, these results are in coherence with Thorne’s (1990) qualitative study, in which mothers with rheumatoid arthritis reported experiencing distress as they feared they were unable to meet their parental expectations, with the added pressure of caregiving responsibilities intensifying feelings of inadequacy and creating family tension. According to Bandura’s self-efficacy theory (1977, 1997) personal context and coping resources affect self-efficacy perception. Since CP implies a personal context that diminishes personal coping capacity, it would be expected that parents with CP would exhibit lower perceived competence than healthy parents.

Parents seem to feel guiltier when they are unable to meet their standards of parental care. This study showed that parents with CP reported significantly higher levels of parental guilt than the group of control parents (although the effect size was small, and these differences should be taken cautiously). In light of the challenges and physical difficulties that parents with CP could have in caring for their children, it makes sense that they would feel they are not meeting their care standards (which is reflected in the lower scores of parental competence compared to the control group) and consequently their levels of parental guilt are higher than those of healthy parents. These findings are consistent with previous qualitative research suggesting that mothers with CP report feeling guilty for not meeting their standards of care due to the impact of pain on their parenting practices, in addition to feeling guilty about the possibility that their children become caregivers, reversing roles (Kristiansen et al., 2012; Meade et al., 2012). Working mothers with conflicts related to work-family guilt have also shown similar results (Maclean et al., 2021). As far as we know, this is the first quantitative study to examine parental competence and parental guilt in a sample of parents with CP and to supply quantitative data supporting previous qualitative data.

In addition, parents with CP showed significantly higher levels of depression than parents without CP. Previous research has shown a strong association between pain and depressive symptoms (Kleykamp et al., 2021; Turesson, 2016) and the results of the present study support those found by Fussner et al. (2018), which showed that parents experiencing CP were more likely to suffer from depression than those who did not. In addition, our results are coherent with the findings of Dany et al. (2016), which showed that patients with CP and children were more likely to suffer from depression than those without children.

In summary, according to our results, parental stress could predict depressive symptoms through its relationship with parental competence. Moreover, parents’ guilty feelings and sense of competence seem to be key variables in explaining depression symptoms both in parents with and without CP. The study of the mental health of parents has been very scarce, focusing instead on the family system or the child. This issue needs to be addressed, especially if the parents suffer from pathologies such as CP since parental psychopathology has been linked to ineffective parenting practices and more child behavior problems (Crum and Moreland, 2017; Haslam et al., 2020; Sutton et al., 2017). A strength of this study is the inclusion of parents with a variety of CP conditions, as well as those without pain in a control group. To the best of our knowledge, this is the first study to test a predictive model of depressive symptoms in parents with CP.

## 5 Conclusion

This study developed a predictive model that fits adequately for both parents with CP and those without pain, suggesting that higher parental stress predicts higher depressive symptoms in parents, and this predictive relationship is mediated by parental competence (the higher the parental stress the lower perceived parental competence). Furthermore, on the one hand, parental competence is related to depressive symptoms in a direct manner, but also, in other hand, there is an indirect relationship mediated by parental guilt (lower competence is related with higher guilt). To the best of our knowledge, this is the first quantitative study to examine parental competence and parental guilt in a sample of parents with CP and the first study analyzing the combined role as mediating factors between parental stress and depression both in CP and in healthy parents. In this vein, our results confirm, with quantitative data, what has been suggested by qualitative studies in CP. In addition, the results extend the findings in other populations of parents under chronic stress conditions to parents with CP. The model tested is not only coherent with the main theories about stress, self-efficacy and depression, but also with previous literature about CP in which the predictive role of self-efficacy on depression is well stablished (Martinez-Calderon et al., 2018).

Furthermore, the levels of the variables analyzed in both samples of parents suggest a worse adjustment to parenthood for parents suffering from CP: higher levels of parental stress, parental guilt and depression, as well as lower levels of parental competence.

Consequently, the results suggest that interventions aimed at improving parental competence and reducing parental guilt could be beneficial to both general populations of parents as well as parents suffering from CP pathologies, thereby reducing levels of depression.

## 6 Implications

As a result of the observed mediation analyses, the implementation of interventions focused on reducing stress, increasing competence and diminishing guilty feelings for parents could reduce levels of depressive symptoms and thereby improve their mental health.

Besides that, parents who experience CP are doubly susceptible to stress since parenthood and coping with pain are both potential sources. Additionally, there is evidence that parental CP may negatively impact the well-being of their offspring, as well as the parent–child relationship. On the one hand, some studies indicate that children whose parents suffer from CP have a higher risk of experiencing pain and poor physical and emotional well-being (Kaasbøll et al., 2012; Higgins et al., 2015). On the other hand, the disabling nature of parental CP increases the risk of role reversal, where children assume responsibilities that they should not have to, resulting in negative consequences for them, which seems to be a concern for parents with CP (Duryea, 2007; Kristiansen et al., 2012; Parton et al., 2022; Umberger, 2014). In addition, lower birth rates have been reported among patients with rheumatoid arthritis, compared to the population without CP (Hunt and Talabi, 2019). These studies conclude that parents sometimes consider having fewer children due to concerns about caring for them as a result of their pain. These findings highlight the importance of addressing mental health comprehensively and providing these patients with appropriate care at the clinical, political, and family levels.

Mental health professionals are required to address potential parenting concerns or difficulties associated with the mentioned variables. Although the results of this study indicate that these interventions would be beneficial for both parents (with and without pain), psychological interventions specifically aimed at parents who suffer from CP may be particularly helpful since they appear to be more likely to experience parental health issues. As interventions based on self-compassion have been shown to reduce parental guilt (Miller and Strachan, 2020; Sirois et al., 2019), it would be interesting to explore the potential applications of these interventions in CP parents. In the same vein, positive parenting programs, such as the ones developed by Tuntipuchitanon et al. (2022) (which include self-control techniques, developing problem-solving skills, increasing the quality of shared time, etc.), have been shown to improve parental competence and consequently parental mental health, as well as their children’s behavioral and emotional problems. CP patients would benefit from psychological interventions aimed at improving their parental skills, since it has been observed that stress levels are reduced and their parental competence is increased (Pinquart, 2018), by focusing on the following areas: highlighting the parental abilities and the skills that can be developed, offering adaptations for those parenting tasks for which they have greater difficulty due to the pain and, if possible, delegating the more difficult parenting tasks to other family members or other supports. Specifically, in this context, it has been shown that social support (e.g., support from another parent or other family member) is associated with lower levels of stress, depression and guilt in paternal CP and other chronic illnesses (Alami, 2023; Evans et al., 2005; Fyrand et al., 1997). The benefits of these interventions would not be limited to the parent but would extend to all family members as well, improving the quality of life of the family system (Scannell, 2021).

Although CP has a high economic impact, little specific policy response has been implemented. Therefore, it will be necessary to give CP a high priority, along with other chronic diseases, and formulate policies that address both the work environment and the family level to mitigate the difficulties that may arise if one of the parents has CP. Support programs have been implemented with successful results in other populations of caregivers, such as people caring for older adults or adults with serious health problems (Chow et al., 2023; Zhai et al., 2023). Such programs usually include one or several of these components: formal social support, skills training, environmental modifications, care management, counseling, and other psychological and multicomponent interventions and could also be useful for parents with CP.

## 7 Limitations and future directions

This study has some limitations which should be considered. First, while we sought to study both fathers and mothers, the majority of the sample was female. Women are more likely to suffer CP than men, which may explain these results in the CP group (Jiménez-Trujillo et al., 2019). Another limitation were the differences found in some sociodemographic variables between CP parents and control parents, with the CP group showing a higher unemployment rate, lower educational level and a higher average age. However, several studies support that the differences found in this study sample could reflect the reality of CP parents (De Sola et al., 2016; Jakobsson et al., 2015; Mills et al., 2019; Mullins et al., 2022). Thirdly, in this study evidence was obtained through a cross-sectional design to generate a predictive model of parental depression valid for both groups of parents. Therefore, for future research, it would be interesting to implement a longitudinal research design to verify the cause-effect relationship between the parental variables analyzed in this study. Also, it would be of interest to determine whether the levels and the relationship of these variables change as their children’s needs change, as it has been shown that parental stress levels fluctuate throughout the child’s development process, with higher levels when children are in early stages of development (0–3 years) (Kanter and Proulx, 2019). In addition, self-report and single-reporter methods were used. Specifically, parents were asked to report variables related to coping with parenthood, but their perceptions could be biased. Particularly, parents who are highly stressed may be more critical of their parenting abilities. Fifthly, other factors not considered in the study may play a role in influencing the associations mentioned. Although it has not been taken into account in this research, future research lines should examine the impact of psychosocial variables such as social support for parents with CP, since there is evidence that this variable can mitigate parental stress and parental guilt levels, but there seems to be a complex relationship between these variables. Parental stress and social support do not appear to be linearly related, and high levels of social support may be associated with increased pain responses (and consequently an increase in dependency) (Evans et al., 2005; Alami, 2023).

## Data Availability

The datasets presented in this study can be found in online repositories. The names of the repository/repositories and accession number(s) can be found at: https://osf.io/8gxhr/.
